# Clinical Characteristics and Risk Factors for *Clostridioides difficile* Infection in the Hematopoietic Cell Transplantation Population

**DOI:** 10.21203/rs.3.rs-4531064/v1

**Published:** 2024-07-10

**Authors:** Abhishek Deshpande, Joseph O'Brien, Betty Hamilton, Matthew Pappas

**Affiliations:** Cleveland Clinic; Cleveland Clinic; Cleveland Clinic

**Keywords:** C. difficile, Hematopoietic cell transplantation, recurrent C. difficile

## Abstract

**Background:**

Hematopoietic cell transplantation (HCT) recipients are at increased risk of developing primary and recurrent *Clostridioides difficile* infection (CDI). The objective of our study was to characterize the risk factors for primary and recurrent CDI in a large cohort of patients hospitalized for HCT.

**Methods:**

We conducted a retrospective cohort study of adults who underwent HCT from 2010–2023 to analyze the epidemiology, timing, and risk factors for CDI. We compared patients who developed CDI with those who did not, controlling for patient demographics, comorbidities, transplant factors, medications, and laboratory values.

**Results:**

Of the 2,725 adults who underwent HCT, 252 (9.3%) developed primary CDI within one-year of transplantation. The incidence was higher among allogenic HCT recipients (17.8%) compared to autologous recipients (4.1%). Independent risk factors for primary CDI included receipt of penicillin antibiotics, prior chemotherapy, and umbilical cord stem cells. Receipt of macrolide antibiotics was an independent risk factor for recurrent CDI, while receipt of autologous HCT was associated with a reduced risk of both primary and recurrent CDI.

**Conclusions:**

CDI presents an early complication after HCT, particularly in allogenic recipients who experience higher incidence rates and severe complications. Early recognition and management of these risk factors are essential to prevent these adverse outcomes.

## INTRODUCTION

*Clostridioides difficile* infection (CDI) is the most common cause of healthcare-associated diarrhea in the United States.^1,2^ Recipients of hematopoietic cell transplantation (HCT) are at increased risk of developing CDI, with approximately 12.5–30% contracting CDI.^3–7^ While the exact mechanism driving this trend is uncertain, prolonged hospitalization, exposure to broad-spectrum antibiotics, immunodeficient state, and loss of gastrointestinal mucosal barrier integrity likely all contribute.^8,9^ The risk of CDI also varies by the type of HCT, from 5–24%^4,5^ among autologous HCT recipients, to 9–34% among allogenic HCT recipients.^10,11^ CDI among the HCT patient population is also associated with worsened clinical outcomes, including sepsis, fulminant colitis, and toxic megacolon.^12^ Previous studies have identified several risk factors for primary CDI among HCT patients, many of which are unique to this population.^6,7^

While CDI therapy is generally effective, 20–30% of HCT patients develop recurrent CDI within 2 weeks of completing treatment.^2^ Fewer studies have investigated risk factors for recurrent CDI in this population. Recurrent CDI carries a high burden of disease, with a 20–30% rate of initial recurrence, 40% second recurrence, and 45–65% third recurrence.^8,13^ Thus, there is a need to better understand the risk factors associated with recurrent CDI in the HCT recipient population using contemporary data. The aim of this study was to characterize the risk factors for primary and recurrent CDI in a large cohort of patients hospitalized for HCT.

## METHODS

### Data source and study cohort:

We assembled a cohort of HCT recipients from the Unified Transplant Database, a prospectively maintained database of autologous and allogenic HCT recipients at the Cleveland Clinic, Cleveland, Ohio, USA. The study was approved by the Cleveland Clinic Institutional Review Board. We included adults (≥ 18 years) with hematological malignancy and/or bone marrow failure syndromes who underwent their first HCT between January 1, 2010 and March 31, 2023. We stratified the cohort into four groups based on their CDI status: patients without CDI, patients with primary CDI, patients with recurrent CDI, and patients with non-recurrent CDI. Patients were considered to have CDI if they had symptoms consistent with CDI, typically new-onset diarrhea (≥ 3 unformed stools/24 hours) and a positive stool test for *C. difficile* as per Infectious Disease Society of America/Society for Healthcare Epidemiology of America (IDSA/SHEA) guidelines.^14^ The institutional laboratory testing algorithm for CDI changed over the study period (2010–2023): CDI was defined as diarrhea and a positive stool test result (positive toxin enzyme immuno-assay (EIA) for *C. difficile* before October 2010 or positive real-time polymerase chain reaction (PCR) toxin B assay after November 2010 to May 2018) or both positive PCR toxin B assay and positive EIA after June 2018. Recurrent CDI was defined as diarrhea and a positive CDI stool test between 2–8 weeks after successful completion of treatment for primary CDI.^14^ Reinfection was defined as CDI occurring > 8 weeks from the first infection.^14^ Severe CDI was defined as a white blood cell (WBC) count > 15,000 cells/mm^3^ and a creatinine level greater than or equal to 1.5 mg/dL, or the presence of hypotension, shock, ileus or toxic megacolon.^14^ Patients with a history of CDI diagnosis within 8 weeks prior to HCT were excluded from the analysis to avoid misclassification of recurrent CDI as primary CDI following HCT.

### Statistical analyses:

We used descriptive statistics to report demographic and clinical characteristics of HCT recipients, stratifying them by CDI status and type of transplant. Continuous variables were summarized using means and standard deviations or medians and interquartile ranges (IQR), and categorical variables as frequencies and percentages. Numerous variables were assessed as risk factors for primary CDI : age, sex, body mass index (BMI), hematopoietic cell transplantation-specific comorbidity index (HCT-CI), total hospital visits in the year preceding HCT admission (number of inpatient days and outpatient visits), insurance (e.g. private, Medicare, Medicaid), race, primary disease, donor type, stem cell source, antibiotic use (e.g. cephalosporins, penicillins, quinolones, clindamycin, macrolides, sulfonamides, tetracyclines), use of gastric acid suppressors, immunosuppressive agents, antidiarrheals, opioids, and total parental nutrition (TPN) (Supplemental Table 1). Medication data were collected as a binary variable indicating whether the patient received at least one dose within 90 days prior to their transplant. Laboratory data were collected on the day of transplant or, for WBC and serum creatinine, the highest value between admission for transplant and the date of transplant. Risk factors were chosen based on expert review and published literature.

Incidence of CDI and trend analysis: The annual incidence rate of CDI was calculated as the number of new CDI cases per year divided by the total number of HCT recipients, reported as percentages. Incidence rates over time were analyzed to assess temporal trends in CDI occurrence. We used a linear regression model to test time trend, with year as the sole independent variable and annual CDI incidence rate as the dependent variable.

Cumulative risk estimation: We estimated the risk of developing CDI within one year of transplantation using cumulative incidence and compared the risks between different transplant types using Gray’s test.

Comparative and risk factor analyses: To identify risk factors for primary CDI, we fit a multivariable regression analysis with primary CDI as the dependent variable. We included patient and treatment-related factors as detailed above and in Table 1. We also examined the duration of medication use within the 90-day pre-transplant period.

## RESULTS

### Study population and CDI rates:

**Figure 1:**
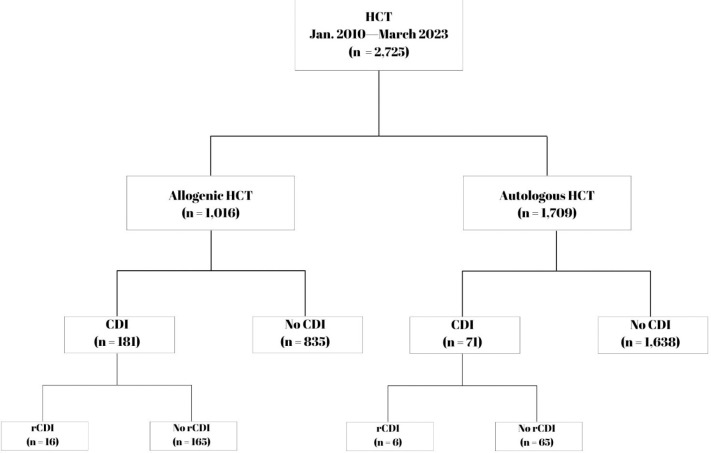
Study population. HCT: hematopoietic cell transplantation; CDI: *C. difficile* infection; rCDI: recurrent *C. difficile* infection.

**Figure 2:**
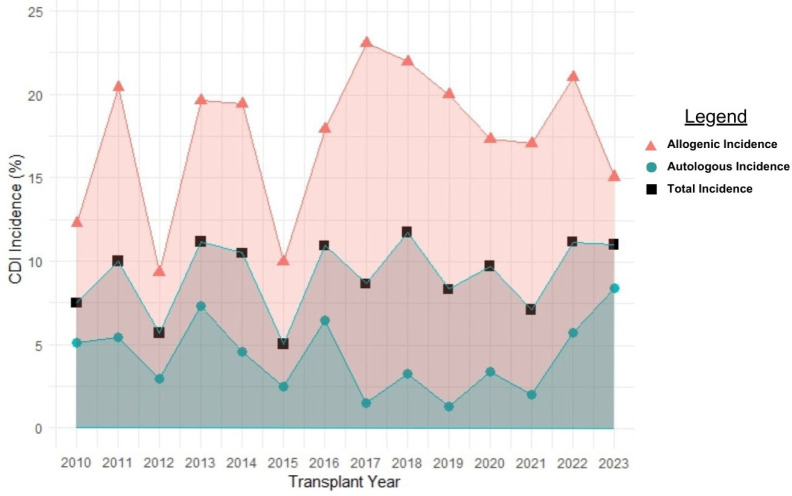
Incidence of primary *C. difficile* infection (CDI) from the day of transplant through one year, stratified by transplant type. The overall CDI incidence increased by an average of 1.4% each year (95% CI: 1.24–1.53%, p < 0.001).

Out of 2,768 patients in the database who completed HCT, we identified 2,725 who met inclusion and exclusion criteria. Of those, 252 (9.3%) patients developed CDI within 1 year from transplant. Of those, 22 (8.7% of the primary CDI population, 0.8% of the entire study population) patients developed recurrent CDI within 1 year. The overall incidence of CDI was 17.8% (181 of 1016 patients) in allogenic HCT recipients and 4.1% in autologous recipients (71 of 1709 patients) (Fig. 1). From 2010 to 2023, the overall CDI incidence increased by an average of 1.4% each year (95%CI: 1.24–1.53%, p < 0.001) (Fig. 2).

**Figure 3:**
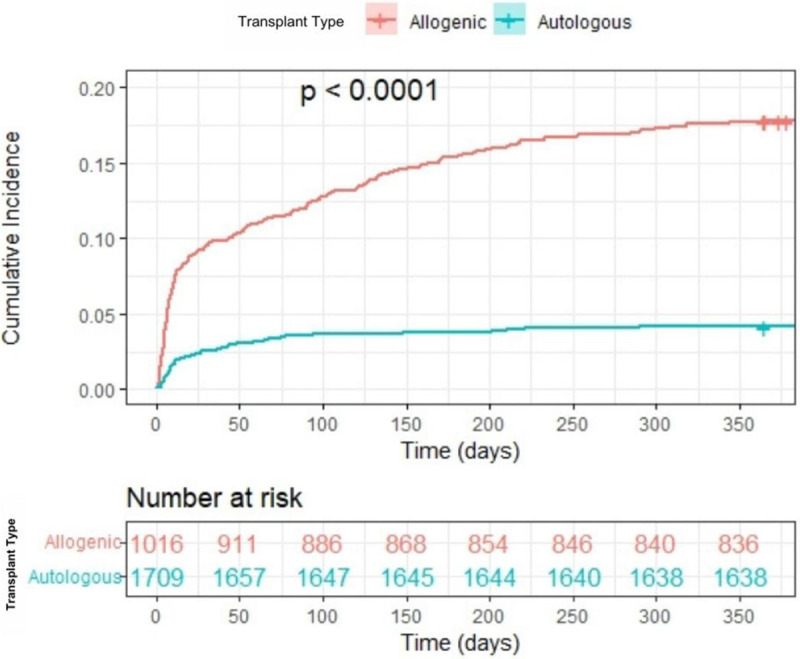
Cumulative incidence of *C. difficile* re-infection (CDI) in the year following transplant, stratified by transplant type. Allogenic HCT recipients were significantly more likely to develop CDI within 1 year following transplantation than recipients of autologous HCT recipients.

Allogenic HCT recipients developed CDI at a median of 21.6 days after transplant (IQR 5.8–120 days). Allogenic HCT recipients were significantly more likely to develop CDI within 1 year following transplantation than autologous HCT recipients (Fig. 3). Autologous HCT recipients developed CDI at a median of 16.8 days after transplant (IQR: 6.9–54.4 days), with 43 (60.5%) diagnosed within 30 days of transplant (Supplemental Fig. 1).

Patients with CDI were similar with respect to age, BMI, sex, and HCT-CI scores to patients who did not develop CDI (Table 1). While CDI and non-CDI patients had similar HCT-CI scores, a greater percentage of patients with CDI had inflammatory bowel disease (6.7% vs. 4.8%) and congestive heart failure (11.5% vs. 8%) compared to those who did not develop CDI. Patients who developed CDI were less likely to have private insurance (64.7%) than patients who did not develop CDI (97.5%). Among patients who developed CDI, the most common primary disease was acute myeloid leukemia (33.7%), followed by multiple myeloma (16.3%) and myelodysplastic syndrome (16.3%).

Patients who developed CDI were more likely to be on penicillins (54.8%) or sulfonamide antibiotics (70.6%) than patients who did not develop CDI (32.2% and 37.2%, respectively). Laboratory findings were similar in patients who developed CDI and those who did not (Table 1). Patients who developed CDI tended to have more severe leukopenia on the day of transplant (820 vs. 1,160 cells/mm^3^) and a lower maximum WBC (4,960 vs. 5,280 cells/mm^3^) during the transplant admission compared to non-CDI patients. Of the patients who developed CDI, most had normal serum albumin levels (median level, 3.5 g/dL; IQR, 3.3–3.7 g/dL) and BUN serum levels (median level, 13 mg/dL; IQR, 9.5–16.5 mg/dL).

### Clinical outcomes

Among HCT recipients who developed CDI, 81.7% developed mild to moderate infection, 2.8% developed severe, uncomplicated infection, and 15.5% developed severe, complicated infection. A sub-analysis of CDI severity among transplant types revealed that allogenic recipients were more likely to develop a severe, complicated infection (OR 4.0; 95%CI, 1.4–11.8) than autologous recipients. Among all HCT recipients who contracted CDI, 72 (28.6%) patients developed sepsis at a higher incidence than patients who did not develop CDI (OR 23.7; 95%CI, 15.7–35.8). This was true among allogenic (OR 12.7; 95%CI, 8.0–20.1) (Supplemental Table 2) and autologous (OR 33.4; 95%CI, 12.7–87.6) recipients (Supplemental Table 3). CDI was associated with an increased risk of developing ileus (OR 15.8; 95%CI, 6.1–41.2), gastrointestinal perforation (OR 9.9; 95%CI, 1.4–70.5), and toxic megacolon (OR 25.0; 95%CI, 4.8–129.6), though base rates of ileus, gastrointestinal perforation, and toxic megacolon were low (Table 1).

Among all HCT recipients who developed CDI, 63 (25.0%) died within 1 year from their transplant, compared to 365 (14.8%) who did not develop CDI. Contracting CDI was associated with a greater risk of 1-year all-cause mortality (OR 1.9; 95%CI, 1.4–2.6). Allogenic recipients were at higher risk of all-cause mortality than their autologous counterparts (OR 5.0; 95%CI, 4.0–6.2), regardless of CDI status. Among patients who contracted CDI, 85 (33.7%) were admitted to the ICU within 1 year of their transplant, compared to 167 (6.8%) of those without CDI (OR 7.0; 95%CI, 5.2–9.5). A sub-analysis of ICU admission stratified on transplant type revealed similar findings among allogenic recipients (OR 11.6; 95%CI, 7.6–17.6) and autologous recipients (OR 21.3; 95%CI, 9.3–48.8) (Supplemental Table 3).

### Risk factors for CDI

In a multivariable model, receipt of several medications remained independently associated with primary CDI among HCT recipients (Table 1), including penicillins (Adjusted Odds Ratio (aOR) 1.5; 95%CI, 1.1–2.1), antidiarrheal medications (aOR 1.4; 95%CI, 1.0–2.0), and previous chemotherapy regimens (aOR 8.4; 95%CI, 3.0–23.7 for receipt of 1–3 previous regimens, aOR 7.1; 95%CI, 2.3–21.7 for receipt of > 3 previous chemotherapy regimens), and stem cells sourced from umbilical cord blood (aOR 2.0; 95%CI, 1.1–3.6). Receipt of autologous transplantation (aOR 0.43; 95%CI, 0.23–0.83) and immunosuppressive medications (aOR 0.23; 95%CI, 0.06–0.88) were independently associated with a lower risk of CDI.

In a second multivariable model using the ‘duration of treatment’ for each medication, antidiarrheal and immunosuppressive medications were no longer associated with CDI. Other significant associations were similar, including receipt of penicillin antibiotics, receipt of 1–3 and > 3 previous chemotherapy regimens, and receipt of umbilical cord stem cells (Supplemental Table 4).

### Recurrent CDI characteristics & outcomes

Among the 252 patients who developed index CDI, 22 (8.7%) developed a first recurrence, 12 (4.8% of index cases; 54.5% of first recurrence cases) developed a second recurrence, and 8 (3.2% of index cases; 75% of second recurrence cases) developed a third recurrence (Supplemental Fig. 2A). Another 39 patients (15.5% of patients with primary CDI) developed infection, a median time to reinfection of 122 days from the index CDI (Supplemental Fig. 2B).

Among 22 patients with recurrent CDI, 12 (54.5%) demonstrated mild to moderate severity, 1 (4.5%) had severe, uncomplicated infection, and 9 (40.9%) developed severe, complicated infection. Recurrent CDI was associated with an increased risk of septic shock (OR 4.42; 95%CI, 1.54–12.68), with 6 (27.3%) of recurrence patients developing septic shock compared to 18 (7.8%) of those who did not have a recurrence. One-year mortality did not differ between patients with recurrence (6 deaths, 27.3%) and patients without recurrent infection (57 deaths, 24.8%).

### Recurrent CDI risk factors

Results of our multivariable regression model of recurrent CDI risk factors are shown in Table 2. Receipt of macrolides prior to the index CDI (aOR 7.3; 95%CI, 1.8–29.2) and allogenic HCT (aOR 31.0; 95%CI, 1.4–731.6) were independently associated with an increased risk of CDI recurrence. A second multivariable regression model was performed to assess risk for medications administered after the index CDI. Macrolide receipt after the index CDI (aOR 6.0; 95%CI, 1.7–21.1) and receipt of allogenic HCT (aOR 19.5; 95%CI, 1.1–365.9) remained independently associated with an increased risk of recurrence.

## DISCUSSION

Our study represents the largest analysis of the epidemiology, timing, and outcomes of primary and recurrent CDI in the HCT population. Approximately 1 in 5 allogenic HCT recipients developed CDI, compared to 1 in 25 patients among autologous HCT recipients. The use of penicillins, prior chemotherapy, and receipt of umbilical cord stem cells were associated with a higher risk for CDI. Among those with CDI, about 1 in 12 developed recurrence, whereas 1 in 4 developed another *C. difficile* infection (CDI > 8 weeks from index CDI). Macrolide use was associated with a higher risk of recurrence, whereas autologous HCT recipients had a lower risk for both primary CDI and recurrent CDI.

In our study, CDI was a relatively common complication of HCT, affecting 9% of all HCT recipients, predominantly among allogenic HCT recipients. The incidence rate was more than four times higher in allogeneic HCT recipients compared to autologous HCT recipients. This higher rate among allogenic HCT recipients is consistent with the published literature^8–10^ and can be attributed to several factors, including prolonged hospital stays, longer antibiotic exposure, and extended periods of reduced immune function. The severity of CDI in our study was similar to findings from previous, smaller studies.^10,15^ In addition to higher rates of CDI, allogenic HCT recipients were more likely to develop a severe, complicated infection than were autologous recipients.

Previous data on the clinical outcomes of CDI among the HCT population have been conflicting.^7,16–18^ In our study, CDI was associated with a nearly 2-fold increase in all-cause mortality 1-year from transplant. This finding is in contrast to some studies^16–18^, which may have been underpowered to detect a statistically significant difference in mortality outcomes, and consistent with other studies.^7,19,20^ Also, in our study, CDI among HCT recipients was associated with an increased risk of adverse outcomes, including ICU admission, sepsis, ileus and gastrointestinal perforation. To the best of our knowledge, ours is the first study to report an increased risk of these adverse outcomes, though others have reported a link between sepsis and risk of developing CDI.^9^

With the largest detailed cohort of HCT recipients our study was powered to investigate multiple risk factors for CDI. While most studies investigated ‘any antibiotic use’, we examined specific antibiotic classes, as well as the ‘duration of use’. This approach addresses a major limitation in most studies examining risk factors for CDI, where a patient who received a single dose of an antibiotic is often equated to a patient who received multiple days of antibiotics. Indeed, our second model showed that when medication duration was accounted for, antidiarrheal medications were no longer associated with CDI. In our analysis, each additional day of penicillin use in the pre-transplant period was associated with a modest increase in risk of CDI. This finding substantiates other analyses^8,15–17,21^ and provides a risk estimation for each additional day of penicillin administration. Unlike some previous studies, we found that certain high-risk antibiotic classes (cephalosporins, clindamycin, sulfonamides, fluoroquinolones) were not associated with an increased risk of CDI after accounting for the duration of antibiotic treatment.

In our study, prior chemotherapy and umbilical cord-derived stem cells conferred an increased risk of CDI. Others have reported prior chemotherapy exposure as an increased risk among allogenic HCT recipients,^8^ and we report similar findings in both allogenic and autologous HCT recipient populations. Umbilical cord stem cells have previously been associated with higher rates of CDI, likely due to reduced T-cell function, immunosuppression, and hypogammaglobulinemia.^16^ Among the 149 patients who received umbilical cord-derived stem cells, 15% developed CDI, which is higher than what others have reported.^16^

Recurrent CDI represents a considerable disease burden, with the first recurrence occurring in about 1 in 12 patients in our study. Many patients had multiple recurrences, and a quarter of patients had *C. difficile* reinfection. In our study, recurrent CDI was not associated with an increased risk of mortality, in agreement with previous studies.^21,22^ We identified macrolide use as an independent risk factor of recurrent CDI. Macrolide use may select for the macrolide-resistant *C. difficile* strain BI/NAP1/027, which has been associated with hospital outbreaks in North America since 2001.^23,24^ Antibiotics alter the native colonic microbiota, making the host susceptible to CDI. Specific antibiotics, like clindamycin, have been shown to select and facilitate infection by strains resistant to that antibiotic.^25,26^ Moreover, the BI/NAP1/027 strain has been shown to be highly resistant to clindamycin, fluoroquinolones, rifampin, and erythromycin.^27,28^ It is possible that macrolide use in the CDI patient population selects for the highly resistant BI/NAP1/027 strain, thereby predisposing them to an increased risk of recurrence. While the number of studies investigating risk factors for recurrent CDI is limited, others have reported the number of antibiotics as an independent risk factor.^21,22^ Here, we examined specific medication class use before and after the index CDI to show macrolide use as an independent risk factor.

Our study has limitations. It was conducted at a single institution, and most patients were White and had private insurance, which may limit the generalizability of our findings. Standalone NAAT for CDI was the diagnostic test of choice at our institution from 2010–2018. Previous studies have suggested that standalone NAAT may have led to an over-diagnosis of CDI.^29^ Our risk factor analysis for recurrent CDI was underpowered to detect some associations, though it remains one of the largest risk factor analysis in the HCT population. Lastly, we included as many risk factors in our model as there were recurrent CDI events, which limits the validation potential of these results in other cohorts.

In conclusion, CDI was a frequent, early complication following HCT with allogenic recipients experiencing higher rates and more severe outcomes compared to autologous recipients. Use of certain antibiotics, prior chemotherapy and receiving umbilical cord stem cells were independent risk factors for developing CDI. CDI was associated with increased mortality and morbidity, and timely identification and management of the risk factors is critical to prevent these adverse outcomes from occurring.

## Figures and Tables

**Table 1:** Demographic and clinical characteristics of HCT recipients at Cleveland Clinic, 2010–2023.

Characteristic	Patients with CDI (n = 252)	Patients without CDI (n = 2473)	AOR (95% CI)	P-value
**Age (yrs)**	65.9 (15.5)	65.7 (15.6)	1.01 (0.99–1.02)	0.13
**BMI (kg/m^2^)**	29.2 ± 5.8	29.8 ± 6.4	1.0 (0.98–1.03)	0.86
**Male sex, no.(%)**	152 (60.3)	1425 (57.6)	1.31 (0.97–1.88)	0.07
Comorbidities, no. (%)				
**HCT-CI**			1.04 (0.99–1.09)	0.28
0–1	70 (27.8)	893 (36.1)	-	-
2	45 (17.9)	422 (17.1)	-	-
≥3	137 (54.4)	1158 (46.8)	-	-
**IBD**	17 (6.7)	119 (4.8)	-	-
**CKD**	8 (3.2)	120 (4.9)	-	-
**CHF**	29 (11.5)	199 (8.0)	-	-
**Total Hospital Visits within 1 year**	19.5 (17)	18 (19)	1.0 (0.99–1.01)	0.19
Prior Chemotherapy Regimens				
None	4 (1.6)	174 (7.0)	Ref	Ref
1–3 regimens	225 (8.9)	2110 (85.3)	8.36 (2.95–23.69)	< 0.0001
> 3 regimens	23 (9.1)	189 (7.6)	7.07 (2.30–21.73)	< 0.0001
Insurance, no. (%)				
Medicare	66 (26.2)	42 (1.7)	-	-
Medicaid	23 (9.1)	19 (0.8)	-	-
Private	163 (64.7)	2412 (97.5)	-	-
Race, no. (%)				
White	220 (87.3)	2141 (86.6)	-	-
Black	20 (7.9)	233 (9.4)	-	-
Other	12 (4.8)	99 (4)	-	-
Hematological Malignancy, no. (%)				
AML	85 (33.7)	337 (13.6)	1.37 (0.66–2.87)	0.40
MDS	41 (16.3)	168 (6.8)	1.47 (0.76–2.85)	0.26
ALL	20 (7.9)	84 (3.4)	1.37 (0.66–2.87)	0.40
Chronic myeloproliferative neoplasm	23 (9.1)	248 (10.0)	0.97 (0.5–1.85)	0.92
Other	23 (9.1)	352 (14.2)	Ref	Ref
Multiple myeloma	41 (16.3)	892 (36.1)	0.97 (0.53–1.79)	0.94
NHL	19 (7.5)	392 (15.9)	0.82 (0.42–1.61)	0.57
Stem Cell Source, no. (%)				
Bone marrow	62 (24.6)	289 (11.7)	Ref	Ref
Peripheral Blood	167 (66.3)	2058 (83.2)	0.90 (0.60–1.33)	0.59
Cord blood	23 (9.1)	126 (5.1)	1.95 (1.07–3.57)	0.029
Graft Type, no. (%)				
Autologous	71 (28.2)	1638 (66.2)	0.43 (0.23–0.83)	0.01
Allogenic	181 (71.8)	835 (33.8)	Ref	Ref
Medications, no. (%)				
Cephalosporins	145 (57.5)	1426 (57.7)	1.06 (0.75–1.51)	0.69
Penicillins	138 (54.8)	797 (32.2)	1.51 (1.13–2.02)	0.01
Quinolones	165 (65.5)	2034 (82.2)	0.84 (0.60–1.19)	0.34
Clindamycin	12 (4.8)	60 (2.4)	1.30 (0.64–2.62)	0.45
Macrolides	27 (10.7)	373 (15.1)	0.80 (0.57–1.13)	0.34
Sulfonamides	178 (70.6)	921 (37.2)	1.49 (0.99–2.22)	0.08
Tetracyclines	8 (3.2)	76 (3.1)	0.92 (0.46–1.84)	0.83
Acid suppression	250 (99.2)	2451 (99.1)	1.35 (0.30–6.11)	0.70
Immunosuppressive agents	248 (98.4)	2463 (99.6)	0.23 (0.06–0.84)	0.03
Antidiarrheal agents	66 (26.2)	401 (16.2)	1.44 (1.03–2.01)	0.03
Opioids	252 (100)	2471 (99.9)	-	-
Laboratory Data				
WBC on Day of Transplant (x10^9^ /L)	0.82 (1.8)	1.16 (2.0)	1.0 (0.99–1.0)	0.46
Max Creatinine during Admission (mg/dL)	0.89 (0.39)	0.86 (0.37)	1.0 (0.99–1.0)	0.68
Max WBC During Admission (x10^9^ /L)	4.96 (4.2)	5.28 (4.0)	1.0 (0.99–1.0)	0.19
Bilirubin on Day of Transplant (mg/dL)	0.45 (0.4)	0.5 (0.3)	1.0 (0.99–1.0)	0.51
Glucose on Day of Transplant (mg/dL)	98.5 (22)	98 (22)	1.0 (0.99–1.0)	0.21
AST on Day of Transplant (units/L)	18 (11.3)	18 (10)	1.0 (0.99–1.0)	0.59
ALT on Day of Transplant (units/L)	20.5 (17.5)	19 (16)	-	-
Albumin on Day of Transplant (g/L)	3.5 (0.4)	3.5 (0.5)	1.0 (0.99—1.0)	0.98
BUN on Day of Transplant (mg/dL)	13 (7)	12 (6)	0.99 (0.97—1.0)	0.43
Clinical Outcomes				
Mortality, no. (%)				
Alive	189 (75.0)	2108 (85.2)	-	-
Death within 30 days	4 (1.9)	27 (1.1)	-	-
Death within 60 days	7 (2.8)	65 (2.6)	-	-
Death within 90 days	11 (4.4)	103 (4.2)	-	-
Death within 1 year	63 (25.0)	365 (14.8)	-	-
**ICU Admission**	85 (33.7)	167 (6.8)	-	-
CDI Severity, no. (%)				
Mild to moderate	206 (81.7)	-	-	-
Severe, uncomplicated	7 (2.8)	-	-	-
Severe, complicated	39 (15.5)	-	-	-
Sepsis	72 (28.6)	41 (1.7)	-	-
Septic Shock	24 (9.5)	15 (0.6)	-	-
Ileus	11 (4.4)	7 (0.3)	-	-
GI Perforation	2 (0.7)	2 (0.08)	-	-
Toxic Megacolon	5 (2.0)	2 (0.08)	-	-

Numerical variables are displayed as mean ± standard deviation or median (IQR), as appropriate. Categorical variables are displayed as number of patients, followed by percentage.

Abbreviations: ALL, acute lymphoblastic leukemia; AML, acute myelogenous leukemia; AOR, adjustedodds ratio; BMI, body mass index; CI, confi dence interval; CML, chronic myelogenous leukemia; CHF,chronic heart failure; CKD, chronic kidney disease; HCT-CI, Hematopoietic Cell Transplantation (HCT)-specifi c Comorbidity Index; IBD, infl ammatory bowel disease; MDS, myelodysplastic syndrome; NHL,Non-Hodgkin lymphoma; WBC, white blood cell.

Recurrent CDI: For patients with CDI, we evaluated risk factors linked to recurrence within – one-year post-transplant. We analyzed risk factors for recurrence and reinfection using appropriate regression models.

All analyses were performed using R statistical software (version 4.1.0), with the *glmnet* package for regression models. Statistical significance was determined with two-sided P-values, considering *P* < 0.05 as statistically significant.

**Table 2:** Risk Factors for recurrent *C. difficile* infection.

Characteristic	First Recurrence Patients (n = 22)	Recovered Patients (n = 230)	AOR^a^ (95% CI)	*P*-value^a^	AOR^b^ (95% CI)	*P*-value^b^
**Age (yrs)**	66.5 (14.8)	66.0 (15.7)	1.01 (0.97–1.06)	0.49	1.03 (0.98–1.08)	0.27
**BMI (kg/m^2^)**	22.2 ± 5.0	29.3 ± 5.9	0.95 (0.86–1.06)	0.39	0.95 (0.86–1.05)	0.34
**Male sex–no.(%)**	12 (54.5)	140 (60.9)	0.60 (0.18–2.06)	0.42	0.48 (0.15–1.52)	0.21
Comorbidities, no. (%)						
HCT-CI	2 (2)	3 (3)	0.78 (0.55–1.09)	0.15	0.81 (0.58–1.11)	0.19
IBD	4 (18.2)	12 (5.7)	-	-	-	-
CKD	2 (9.1)	6 (2.6)	-	-	-	-
CHF	3 (13.6)	26 (11.3)	-	-	-	-
Race, **no. (%)**						
White	21 (95.5)	230 (86.5)	-	-	-	-
Black	1 (4.6)	19 (8.3)	-	-	-	-
Other	0 (0)	12 (5.2)	-	-	-	-
Insurance, no. (%)						
Medicare	8 (36.4)	58 (25.2)	-	-	-	-
Medicaid	1 (4.6)	22 (9.6)	-	-	-	-
Private	13 (59.1)	150 (65.2)	-	-	-	-
Hematological Malignancy, no. (%)						
AML	6 (27.3)	79 (34.3)	2.55 (0.18–35.5)	0.49	2.06 (0.15–29.30)	0.59
MDS	3 (13.6)	38 (16.5)	2.65 (0.15–4.87)	0.51	1.27 (0.07–21.1)	0.87
ALL	2 (9.1)	18 (7.8)	4.64 (0.20–10.8)	0.33	5.31 (0.24–11.6)	0.29
Chronic myeloproliferative neoplasm	4 (18.1)	19 (8.3)	11.0 (0.60–20.0)	0.105	10.5 (0.59–18.7)	0.11
Other	1 (4.5)	22 (9.6)	Ref	Ref	Ref	Ref
Multiple myeloma	3 (13.6)	38 (16.5)	27.0 (0.84–86.9)	0.062	22.1 (0.60–81.5)	0.09
**NHL**	3 (13.6)	16 (7.0)	39.7 (0.93–169.2)	0.055	47.3 (0.86–260)	0.06
Stem Cell Source, no. (%)						
Bone marrow	2 (9.1)	60 (26.1)	Ref	Ref	Ref	Ref
Peripheral Blood	18 (81.8)	149 (64.8)	1.81 (0.98–1.84)	0.07	6.1 (0.8–41.95)	0.07
Cord blood	2 (9.1)	21 (9.1)	5.38 (0.52–56.1)	0.16	8.91 (0.76–105.2)	0.08
Transplant Type, no. (%)						
Autologous	6 (27.3)	65 (28.3)	Ref	Ref	Ref	Ref
Allogenic	16 (72.7)	165 (71.7)	31.04 (1.37–731.58)	0.03	19.48 (1.04–365.85)	0.047
Laboratory Data						
WBC on Day of Index CDI diagnosis (x10^9^ /L)	4.75 (3.19)	2.45 (5.74)	1.1 (0.99–1.22)	0.07	1.06 (0.96–1.17)	0.26
Max Creatinine during Admission (mg/dL)	1.03 (0.69)	0.93 (0.42)	1.88 (0.97–3.65)	0.58	1.55 (0.84–2.86)	0.16
Max WBC During Admission (x10^9^ /L)	5.8 (4.28)	4.4 (7.23)	1.0 (0.99–1.0)	0.16	1.0 (0.99–1.0)	0.31
Bilirubin on Day of Index CDI diagnosis (mg/dL)	0.6 (0.6)	0.5 (0.4)	1.06 (0.88–1.29)	0.53	1.06 (0.87–1.29)	0.56
Glucose on Day of Transplant (mg/dL)	109 (33.5)	105 (33)	0.99 (0.98–1.01)	0.35	0.99 (0.98–1.01)	0.48
AST on Day of Index CDI diagnosis (units/L)	24 (18.2)	20 (15)	1.0 (0.99–1.0)	0.91	1.0 (0.99–1.0)	0.78
Albumin on Day of Index CDI diagnosis (g/L)	3.5 (0.8)	3.4 (0.8)	2.0 (0.65–6.13)	0.23	1.91 (0.63–5.75)	0.25
BUN on Day of Index CDI diagnosis (mg/dL)	16 (10)	14 (12)	0.96 (0.88–1.04)	0.33	0.97 (0.90–1.05)	0.51
Medications Prior to Index CDI, no. (%)						
Cephalosporins	10 (45.4)	121 (52.6)	1.04 (0.31–3.46)	0.95	-	-
Penicillins	16 (72.3)	171 (74.3)	0.95 (0.20–4.53)	0.95	-	-
Quinolones	14 (63.6)	133 (57.8)	1.96 (0.52–7.41)	0.32	-	-
Clindamycin	1 (4.5)	8 (3.5)	2.4 (0.18–3.17)	0.51	-	-
Macrolides	8 (36.4)	32 (13.9)	7.25 (1.80–29.2)	0.005	-	-
Sulfonamides	12 (54.5)	155 (67.4)	0.70 (0.15–3.38)	0.66	-	-
Tetracyclines	1 (4.5)	5 (2.2)	0.66 (0.46–9.57)	0.17	-	-
Acid suppression	19 (86.4)	214 (93.0)	1.11 (0.73–1.69)	0.94	-	-
Immunosuppressive agents	20 (90.9)	228 (99.1)	0.02 (0.001–2.0)	0.08	-	-
Antidiarrheal agents	9 (40.9)	94 (40.9)	1.16 (0.29–4.11)	0.82	-	-
Opioid	20 (90.9)	220 (95.7)	-	-	-	-
TPN	1 (4.5)	3 (1.3)	-	-	-	-
Medications After Index CDI, no. (%)						
Cephalosporins	5 (22.7)	61 (26.5)	-	-	0.58 (0.13–2.21)	0.45
Penicillins	12 (54.5)	162 (70.4)	-	-	0.36 (0.08–1.54)	0.16
Quinolones	6 (27.3)	78 (33.9)	-	-	1.26 (0.35–4.47)	0.72
Clindamycin	0 (0)	6 (2.6)	-	-	-	-
Macrolides	11 (50.0)	53 (23.0)	-	-	6.03 (1.72–21.13)	0.005
Sulfonamides	10 (45.5)	139 (60.4)	-	-	0.81 (0.20–3.35)	
Tetracyclines	0 (0)	7 (3.0)	-	-	-	-
IV Metronidazole	4 (18.2)	34 (14.8)	-	-	-	-
Acid suppression	16 (72.7)	168 (73.0)	-	-	1.87 (0.48–7.27)	0.37
Immunosuppressive agents	18 (81.2)	201 (87.4)	-	-	0.65 (0.11–3.78)	0.63
Opioid	18 (81.2)	192 (83.5)	-	-	-	-
TPN	0 (0)	7 (3.0)	-	-	-	-
IV Vancomycin	3 (13.6)	24 (10.4)	-	-	-	-
Oral Vancomycin	11 (50)	100 (43.5)	-	-	-	-
**Oral Metronidazole**	0 (0)	1 (0.4)	-	-	-	-
**Fidaxomicin**	3 (13.6)	5 (2.2)	-	-	-	-
Outcomes, no. (%)						
Death within 1 year	6 (27.3)	57 (24.8)	-	-	-	-
Sepsis	8 (36.4)	64 (27.8)	-	-	-	-
Septic Shock	6 (27.3)	18 (7.8)	-	-	-	-
Ileus	1 (4.5)	10 (4.3)	-	-	-	-
GI Perforation	0 (0)	2 (0.9)	-	-	-	-
Toxic Megacolon	1 (4.5)	4 (1.7)	-	-	-	-

Recurrent *C. difficile* infection was defined as the presence of diarrhea and a positive stool test within 2–8 weeks after completion of treatment for the index infection. Laboratory data are reported from the day of the index *C. difficile* infection. Medication data are reported as either receipt of medication 90 days before or 90 days after the index *C. difficile* infection. Numerical variables are displayed as mean ± standard deviation or median (IQR), as appropriate. Categorical variables are displayed as number of patients, followed by percentage.

a Regression Analysis including medications administered within 90 days prior to index *C. difficile* infection.

b Regression Analysis including medications administered within 90 days after index *C. difficile* infection.

Abbreviations: ALL, acute lymphoblastic leukemia; AML, acute myelogenous leukemia; AOR, adjusted odds ratio; BMI, body mass index; CI, confidence interval; CML, chronic myelogenous leukemia; CHF, chronic heart failure; CKD, chronic kidney disease; HCT-CI, Hematopoietic Cell Transplantation (HCT)-specific Comorbidity Index; IBD, inflammatory bowel disease; MDS, myelodysplastic syndrome; NHL, Non-Hodgkin lymphoma; TPN, total parental nutrition; WBC, white blood cell.

## Data Availability

The datasets generated during and/or analysed during the current study are available from the corresponding author on reasonable request.
